# Commentary on “Association of Serum Total Bilirubin to Cholesterol Ratio With Progression of Chronic Kidney Disease in Patients With Type 2 Diabetes: A Retrospective Cohort Study”

**DOI:** 10.1111/1753-0407.70129

**Published:** 2025-07-21

**Authors:** Tinghua Zhang

**Affiliations:** ^1^ Department of Transfusion The Central Hospital of Huaihua City Huaihua Hunan China; ^2^ Department of Clinical Laboratory The Central Hospital of Huaihua City Huaihua Hunan China

**Keywords:** chronic kidney disease, cohort study, total bilirubin to total cholesterol ratio, type 2 diabetes

**Summary**

- Chen et al. have highlighted the potential of the TBIL/TC ratio as a biomarker for CKD progression in type 2 diabetes.
- Future studies should aim to:
  ○ Validate these findings in ambulatory cohorts using KDIGO‐endorsed sustained eGFR decline criteria.
  ○ Integrate time‐varying adjustments for nephroprotective medications and account for genetic and environmental confounders.
  ○ Report absolute risks and conduct decision‐curve analyses to better evaluate the clinical utility of the TBIL/TC ratio.

To the Editor,

We have read with interest the recent study by Chen et al. published in the Journal of Diabetes [1], which investigates the potential association between the serum total bilirubin to total cholesterol (TBIL/TC) ratio and the progression of chronic kidney disease (CKD) in patients with type 2 diabetes. While the study provides valuable insights into a potential biomarker, several methodological warrant attention to ensure robust and reliable interpretation of the findings.

1. Outcome Definition and Potential Misclassification

The primary outcome of CKD progression, defined as a ≥ 25% decline in estimated glomerular filtration rate (eGFR) combined with a drop in KDIGO category, may be susceptible to misclassification. The reliance on a single baseline eGFR measurement may fail to account for intraindividual variability. Acute illness [2] or dehydration [3] during hospitalization can transiently reduce eGFR levels, thereby inflating the observed rates of CKD progression. According to KDIGO guidelines, sustained CKD progression should be confirmed by serial eGFR measurements over a defined period to minimize such transient effects [4].

2 Inadequate Adjustment for Nephroprotective Medications

Although sensitivity analyses were conducted for SGLT2 inhibitors (SGLT2i) and glucagon‐like peptide‐1 receptor agonists (GLP‐1RA), these critical confounders were excluded from the primary multivariable model presented. Given the well‐documented renoprotective effects of these medications, their omission likely introduces bias in the observed hazard ratios. Furthermore, the study did not account for time‐varying medication use, such as initiation or discontinuation during follow‐up, which could potentially attenuate the true association between the TBIL/TC ratio and CKD progression.

3 Threshold Effect and Clinical Utility

The restricted cubic spline analysis identified a threshold effect at a TBIL/TC ratio of 0.25%. However, subsequent analyses dichotomized the ratio, which discards valuable information and oversimplifies the dose–response relationship. Moreover, the clinical relevance of this specific threshold remains unclear, as the study did not report absolute risk differences across quartiles. This omission limits the translational impact of the findings and hampers the assessment of the clinical utility of the TBIL/TC ratio as a biomarker.

4 Generalizability and Selection Bias

The study cohort consisted of hospitalized patients with relatively high glycated hemoglobin (HbA1c) levels (mean 8.76%) and excluded individuals with eGFR < 15 mL/min/1.73 m^2^. This selection bias restricts the applicability of the findings to stable outpatients or those with advanced CKD. Additionally, 49% of the initially screened patients were excluded due to missing follow‐up eGFR data, which introduces a potential risk of attrition bias and limits the generalizability of the results.

5 Unaddressed Residual Confounding

Several key factors influencing bilirubin levels and lipid metabolism were not measured or adjusted for in the study. Notably, hemoglobin, which affects bilirubin metabolism [5], was not included in the analysis. Furthermore, longitudinal changes in body mass index (BMI), blood pressure, or albuminuria during follow‐up were also not accounted for. These unmeasured confounders may influence the observed association between the TBIL/TC ratio and CKD progression.

## Conclusion

Chen et al. have highlighted the potential of the TBIL/TC ratio as a biomarker for CKD progression in type 2 diabetes. However, the issues of outcome misclassification, medication confounding, and oversimplified threshold analyses necessitate cautious interpretation of their findings. Future studies should aim to: (1) Validate these findings in ambulatory cohorts using KDIGO‐endorsed sustained eGFR decline criteria; (2) Integrate time‐varying adjustments for nephroprotective medications and account for genetic and environmental confounders; (3) Report absolute risks and conduct decision‐curve analyses to better evaluate the clinical utility of the TBIL/TC ratio. Addressing these limitations will help clarify whether the TBIL/TC ratio offers incremental value beyond established renal risk markers.

## Author Contributions

Tinghua Zhang: wrote, reviewed, and edited the manuscript. Additionally, he conceptualized the study concept and design.

## Conflicts of Interest

The author declares no conflicts of interest.
